# Evaluation of a Specialist Nurse-Led Post-Coronary Heart Disease Support Program: A Prospective Pre–Post Interventional Study

**DOI:** 10.3390/healthcare12242497

**Published:** 2024-12-11

**Authors:** Ilias Molos, Christos Kleisiaris, Athina Patelarou, George Kasimis, Savvato Karavasileiadou, Alaa Alanazi, Poulimenos Leonidas, Nikolaos Bakalis

**Affiliations:** 1 Department of Nursing, Faculty of Health and Rehabilitation Sciences, University of Patras, 26504 Patras, Greece; up1089361@upatras.gr (I.M.); nikosbakalis@upatras.gr (N.B.); 2 Department of Nursing, University of Thessaly, 41500 Larissa, Greece; kleisiaris@uth.gr; 3 Department of Nursing, Hellenic Mediterranean University, 71410 Heraklion, Greece; apatelarou@hmu.gr; 4 Department of Cardiology, Aristotle University of Thessaloniki, 54124 Thessaloniki, Greece; gkasimis@auth.gr; 5 Department of Community and Psychiatric Mental Health Nursing, College of Nursing, Princess Nourah Bint Abdulrahman University, Riyadh 11671, Saudi Arabia; skaravasileiadou@pnu.edu.sa; 6 Department of Medical Surgical Nursing, College of Nursing, Princess Nourah Bint Abdulrahman University, Riyadh 11671, Saudi Arabia; 7 Department of Cardiology, Asklepieion General Hospital, 16673 Athens, Greece; leonp@otenet.gr

**Keywords:** coronary artery disease, secondary prevention, anxiety, dietary habits

## Abstract

Introduction: The impact of multidisciplinary supervised cardiac rehabilitation programs on reducing the risk of having heart problems in the future is well documented. However, little is known about nurse-led post-coronary heart disease (post-CHD). Purpose: Our aim was to evaluate the effectiveness of an educational and counseling-structured nurse-led post-CHD support program by assessing the prediction of psychological, behavioral and dietary variables in relation to adherence to a care plan in a single hospital in Athens (Greece). Method: A prospective follow-up comparative approach in a single group of CHD patients was applied. The structured nurse-led program included eight topics (management of anxiety, physical activity, dietary habits, weight control, smoking cessation, alcohol moderation, family engagement and adherence to a care plan). Participants received tailored nursing interventions focused on psychological and behavioral needs and dietary habits by a CHD-specialized nurse based on patients’ medical prescriptions and/or instructions. A modified clinical assessment questionnaire by the European Society of Cardiology was applied to identify pre–post clinical baseline measurements. A nurse-led post-coronary Heart Disease Support Program was evaluated by assessing the predictivity (effect) of specific interventions on adherence to a care plan by employing a logistic regression beta coefficient (Generalized Estimating Equations model). Results: The sample consisted of 275 patients (66.2% male), with a mean age of 68.5 ± 12.5 years old. CHD patients presented significantly lower anxiety rates (54.27 [1st m] vs. 49.63 [2nd m], *p* < 0.05). In addition, significant differences were observed between the first and the second measurements of total cholesterol (163.27 [1rst m] vs. 134.44 [2nd m], *p* < 0.001), BMI (obesity) (33.69 [1rst m] vs. 32.79 [2nd m], *p* < 0.001), smoking (42.18 [1rst m] vs. 22.55 [2nd m], *p* < 0.001) and adherence to a care plan (78.90 [1rst m] vs. 89.10 [2nd m], *p* < 0.001). A Generalized Estimating Equations model revealed that participants with higher levels of anxiety showed significantly lower adherence to a care plan (beta −0.10, *p* < 0.001) and those with family history of CHD (beta −0.71, *p* = 0.04) in comparison to those with no CHD history. No significant differences were observed in the predictive variables of smoking (beta 0.08, *p* = 0.69), alcohol consumption (beta 0.09, *p* = 0.79) and family engagement (beta −0.11, *p* = 069) with respect to adherence to a care plan, even after adjusting for age, sex and history of CHD. Conclusion: Our findings indicate that the nurse-led post-CHD support program was found to be partially feasible and effective in our single-group study, emphasizing the need for effective training and retention strategies to enhance the specialization of nurses providing post-CHD care and support.

## 1. Introduction

Coronary Heart Disease (CHD) is a major cause of loss of healthy life years all over the world and data for the year 2021 indicate that there were 9.44 million deaths and 185 million disability-adjusted life years lost due to CHD [1].

Secondary prevention is the prevention of damage and progression of an established disease; such prevention measures are adopted to avoid cerebrovascular and peripheral arterial disease [2]. The overall objective of nursing interventions in CHD is to lower the risk of complications and death [3]. Therefore, nurses may help a CHD patient by administering medications to lower cholesterol levels and control hypertension, among other risk factors associated with the disease [4]. Specialist nurses in post-CHD cases play a key role in such prevention since they need to care for their patients and promote healthy lifestyles and choices. In addition, once CHD damage has occurred, the risk of relapse can be decreased by providing a supportive environment to patients and their families with the contribution of a specialist nurse in post-CHD in the context of secondary prevention. Within a broader framework, the specialist nurse in post-CHD not only achieves the physical health of CHD patients but also plays a critical role as an agent for their behavioral progress and improvement in their psychological state [5].

It has been suggested that various behavioral risk factors such as smoking, physical inactivity, weight management, alcohol consumption [6], family engagement [7] and adherence to a care plan [8] are known to be important risk factors for CHD. Most behavioral risk factors are potentially modifiable ones [6]. Nurses may decrease premature morbidity and CHD mortality by controlling these modifiable behavioral risk factors [9]. In addition, one of the main goals of a specialist nurse in post-CHD care is to deter psychological risk factors (e.g., aggravating anxiety) that may cause further complications. This can be achieved through education and information teaching, so that salient needs are covered. It is, therefore, crucial that patients with CHD and their families are not neglected. Instead, they must receive appropriate training and support to prioritize their needs. Anxiety symptoms, particularly panic attacks, are independently and significantly associated with an increased overall risk of developing CHD over ten years [10]. Further specialized studies on the aftermath of anxiety are in progress to investigate the mechanisms associated with particular types of anxiety caused by CHD, as well as possible interventions to mitigate these processes. For this purpose, the contribution of nurses is essential, elevating the importance of the role of nurses and having positive benefits for CHD patients [11].

It is well known that dietary preventive strategies such as calorie control and diets that are nutrient-rich and low in fatty acids, cholesterol, sodium and added sugars are reducing the progression of disease and preventing the recurrence of clinical events in patients with CHD [12]. These dietary patterns promote the consumption of vegetables, fruits and whole grains and emphasize unsaturated fats from nuts, seeds and plant oils. Reduced-fat milk products, seafood and lean animal proteins are also encouraged. Hence, these dietary patterns can reduce CHD risk by lowering serum glucose, total cholesterol, High Density Lipoprotein-Low Density Lipoproteins (HDL-LDL) and triglycerides [13]. In addition, studies have found a positive correlation between BMI and serum uric acid (SUA), liver enzymes (AST, ALT) and urea [14].

Most importantly, the significant role of specialist nurses in post-CHD supportive care is extensively recognized worldwide. Evidence from Australia and North Korea has shown that secondary prevention programs assist in managing risk factors, preventing disease progression, minimizing complications and reducing hospital readmissions [15]. In Europe, a similar secondary prevention program in Scandinavia, conducted in patients’ homes, revealed that a tailored, nurse-led cardiac rehabilitation program can improve risk factor management in post-CHD patients [16]. These programs are highly recommended for all CHD patients to restore their quality of life, maintain and improve their functional capacity and prevent disease recurrence [17]. Therefore, the importance of creating new concepts and structures regarding the contribution of nurse consultants is highlighted.

Undergraduate education in Greece lasts 4 years (eight semesters, 240 ECTS), leading to a BSC degree. All nursing students follow a combination of theoretical and hospital-based clinical training. Training of nurses in Greece conforms to EU standards for mutual recognition of qualifications according to European Community directives regulating the free movement of European health professionals [18]. Most European countries have the same common structure as Greece; however, different countries have different methods regarding nurses’ qualifications, such as the United States [19] and China [20].

Nursing in Greece is regulated by the Hellenic Regulatory Board of Nurses (HRBN), which was established in 2004 under Law 3252/2004. All nurses who have graduated from universities must be licensed to the HRBN. The vast majority of nurses in Greece work in hospitals, following the emphases given by the state, and in treatment (tertiary care) rather than in prevention (community care). This reflects the philosophy that care is based on medical orientation and thus limits the multi-dimensional role of nurses in patient care. In addition, the nursing working method at hospitals is usually task-oriented and therefore holistic nursing care is hardly provided. These are the main differences from other countries where nurses have a more independent role in patient care based on the nursing process [21].

It is worth mentioning that there are no advanced practice nursing roles in Greece. Although there are ten nursing specialties at the post-registration level provided by the Ministry of Health (free of charge), all of them require only general expertise (like pathology, surgical, community, etc.) and are not specialized (cardiology, oncology, etc.). Nevertheless, there are MSc specialization courses in clinical areas related to nursing science provided by universities. The lack of specialization training and programs is related to the medical model described above, where the majority of care and treatment decisions are made by doctors, leaving only a very small opportunity for other healthcare professionals to participate in the provision of multidisciplinary care [19]. This is different from other countries where nurses obtain specialization along with their graduation from university, providing advanced programs in relation to specific expert roles or through postgraduate (MSc) courses.

Importantly, nurse-led cardiac rehabilitation with respect to secondary prevention with a focus on counseling and health education is a vital component of a comprehensive approach to treating CHD [22]. Nevertheless, in Greece, post-CHD supportive care is largely provided and managed by a responsible cardiologist and less by a multi-disciplinary team. As a result, CHD patients do not receive a holistic approach, which is also true of their relatives [23]. It is obvious that nurses’ roles are limited to basic (routine) nursing tasks and duties, thus minimizing the possibility of advanced specialization in post-CHD supportive care.

Most importantly, to the best of our knowledge, no studies are available in Greece on expanding the role of nurses in post-CHD care. Within a broader framework, nurses caring for CHD patients would benefit from information regarding the secondary preventive interventions that a specialized CHD nurse could provide and further understand whether these patients might benefit from success in maintaining disease control and making positive changes in their self-management behaviors.

Therefore, this survey investigated the existing gap regarding the factors that may predict adherence to care plans in post-CHD supportive care, mainly to draw attention to the effectiveness of a nurse-led post-CHD support program.

## 2. Materials and Methods

### 2.1. Study Design

This study was based on a prospective, cross-sectional follow-up approach based on a nurse-led modified questionnaire that collected demographic, health history and laboratory data of patients. A single-group clinical study design was chosen and implemented to ensure that all participants would receive the same intervention over time, including counseling, psychological support, drug monitoring and delivery methods, in order to explain why the outcome (effectiveness of a nurse-led post-CHD support program) was more effective with that intervention. Recognizing that control groups play a fundamental role as they serve as a baseline for determining the effectiveness of nurse-led post-CHD support programs, a control study was not feasible due to the fact that the specific protocol used in the Department of Cardiology is controlled by cardiologists and not by nurses [24,25]. Consequently, to avoid methodological issues such as sampling and/or survivorship bias (selection bias), we focus on nursing interventions that are managed and monitored mainly by nurses.

A follow-up approach was utilized, which provided two snapshots of the patients’ physical, behavioral and psychometric measurements before the intervention of a specialist nurse in post-CHD and at three-month follow-up after the start of the intervention. Also, the predetermined procedure was implemented in the same mode two times. Specifically, our follow-up approach was based on both the American Heart Association/American College of Cardiology guidelines for CHD [26], and the guidelines from the Greek Ministry of Health recommendations (3–6 months follow-up). Furthermore, the most recent recommendations suggest that the 6-month follow-up may apply to 3-month follow-up [27].

### 2.2. Sampling and Research Procedures

Coronary heart disease patients were randomly selected according to patients’ identifying numbers, as assigned in the cardiology clinic database; patients with CHD took part in the research twice, with an interval of 3 months.

The participants were first informed about the step-by-step procedure of the research by the main researcher (I.M.). Before its implementation, the participants gave their written informed consent after having been fully informed by the main researcher (nurse consultant) that their participation was voluntary and at any time could contact the nurse if they had any inquiries or wished to withdraw from the study. Then, the modified questionnaire was filled in. Finally, before the participants’ appointments ended, the main researcher (nurse consultant) implemented the nursing program by communicating once per month with the participants.

In the present research, the nursing program content was based on a literature search and theoretically supported by Callista Roy’s adaptation model [28]. The tailored nurse-led program modified eight topics [29], focusing more on nursing interventions that could be managed and monitored accurately, which are as follows: (1) management of anxiety, (2) physical activity monitoring, (3) retention of a healthy diet (dietary factors), (4) weight controlling, (5) smoking cessation, (6) alcohol consumption (how much/often drink), (7) family engagement and (8) adherence to the care plan. The nurse-led program was based on ESC Guidelines [30].

Callista Roy’s adaptation model illustrates that throughout the nursing process, the nurse and other healthcare professionals should adapt the nursing care plan based on the patient’s progress toward health [28]. The principle supports the idea that the patient can handle the changes in their environment effectively and then influence it. This nursing program is novel, following the six-step nursing process of Callista Roy’s Adaptation Model. The first part of the demographic data of the questionnaires was primarily organized in agreement with the first and second levels of Callista Roy’s Adaptation model. It had as its target addressing CHD patient behavior and stimuli with patient psychology in mind (usage of Zung Self-Rating Anxiety Scale—SAS) and their positive mood and cooperation throughout the program. Afterwards, given that each patient’s diagnosis was known (CHD—third level), the assignment set goals for CHD patients’ health (fourth level). These goals took two points into account: first, the capability of patients and their families to follow the program, and second, the interventions to make these goals feasible (fifth level). Finally, at the sixth level, the project evaluated the result to determine if the goals were met and, at the same time, if there was acceptance of the procedure by the CHD patients and their families.

It is worth noting that Callista Roy’s adaptation model was enriched because the adaptation was the first to exclusively focused on CHD patients and their families, aiming at extracting more specialized and scientific results around them. Last, but not least, the adaptive responses may vary by individual and may take a longer time compared to others. In the present study, we did not have a variance between the study subjects’ characteristics and the resulting outcomes of the program.

### 2.3. Research Instruments

The questionnaire used for the present study consisted of two parts. The questionnaire from the cardiovascular protection clinic at the cardiology department of the “Asklepieion Voula” General Hospital was used for the first part. The questionnaire was created based on standards set out in the European Society of Cardiology (ESC) set. The questionnaire was modified, and some parts, primarily medical ones like electrocardiogram, exercise stress test and ultrasound, were omitted—this questionnaire modification aimed to promote the nursing approach. The questionnaire used in the clinic consisted of (a) demographic data, (b) clinical information about the patient’s and family’s medical history, (c) clinical examination (height, weight, waist circumference and BP), and (d) a blood sample was obtained for the necessary laboratory tests, consistent with CHD (AST, ALT, serum potassium and sodium, total cholesterol) [31]. In addition, a record was made of the treatment the patient received up to his first visit so that any change in medication based on medical instructions could be assessed at the end of his second visit. For the second part, the Greek version of Zung’s Anxiety Self-Assessment Scale (SAS) was employed to measure patients’ anxiety.

The Zung Self-Rating Anxiety Scale (SAS) is a 20-item self-report rating scale developed to measure anxiety levels based on scores in four subtests/domains: (a) Cognitive, (b) Autonomic, (c) Motor, and (d) Central Nervous System symptoms. Each participant was asked to choose, for each of the 20 items, from a 4-point Likert-type scale (1 = never or rarely, 2 = sometimes, 3 = several times, 4 = many times/always), the answer that corresponded best with their condition. The total score is determined as follows: <50 is considered to correspond to normal limits without evidence of anxiety, values of 50–59 to the presence of minimal to mild anxiety, values of 60–69 to moderate to severe anxiety, and values of >70 to severe to non-curable anxiety [32].

After the modifications were made, an expert panel was used to generate new ideas for the program and discuss risk factors that researchers should focus on. The panel consisted of three cardiologists with postgraduate degrees and clinical experience who refined the items to reduce their number. When the expert panel agreed on the modified questionnaire, a pilot study that enrolled 25 patients was conducted. A power analysis was performed to calculate the required sample size. At least 275 individuals were needed for a smaller effect size (of the order of 0.15) to have a power of at least 80% [33].

### 2.4. Evaluation of Nurse-Led Post-CHD Support Program

The effectiveness of our structured nurse-led post-CHD supportive program was evaluated by assessing the influence (prediction) of the following variables: Psychological: anxiety; Behavioral: physical activity, BMI (weight loss), smoking cessation, alcohol consumption and family engagement regarding adherence to the care plan); we set “adherence to care plan” as the dependent variable. To control their independent association, we adjusted for potential confounding effects: age, sex, history of CHD and diagnosis of dyslipidemia. We also separately examined the impact of dietary factors on adherence to the care plan by assessing possible changes in laboratory results (Urea, Serum Glucose, Uric Acid, Total Cholesterol, HDL, LDL, Triglycerides, AST, and ALT) between the baseline and the follow-up measurements.

Adherence to the care plan was defined by the positive responses (Yes) to five control variables related to educational interventions as follows: Smoking: have you quit smoking? (Yes vs. No); Alcohol Consumption: did you reduce drinking? (Yes vs. No); Weight: did you lose weight? (Yes vs. No); Physical Activity: did you walk at least 3 hrs per week? (Yes vs. No); Family engagement: did your relatives support you? (Yes vs. No).

### 2.5. Ethical Considerations

The research was applied in full compliance with the new General Data Protection Regulation (GDPR) [EU 2016/679] and the Declaration of Helsinki was taken into account, which constitutes a key part of the overall process of evaluating research proposals in agreement with the fundamental principles and standards of research ethics [34]. Furthermore, permission was granted by the Ethics and Deontology Committee of the University of Patras (IRB: Pr. No. 8088/11 July 2021) and by the scientific council of the Asklepieion Voula General Hospital (Protocol number 2005/12 February 2021).

### 2.6. Statistical Analysis

Statistical analyses were performed using IBM SPSS version 28. A statistical significance of *p* = 0.05 was used. Differences in patient measurements before and after the intervention were determined using Paired-Samples *t*-tests. For post hoc comparisons, the Bonferroni test was utilized. To evaluate the effectiveness of our nurse-led post-CHD support program, we examined factors associated with patient adherence to the care plan between the first and second measurements by applying a logistic regression beta coefficient, and particularly a Generalized Estimating Equations (GEEs), model to estimate the parameters of a generalized linear model with a possible unmeasured correlation between observations from different time points.

## 3. Results

Table 1 shows the demographic characteristics and the medical history of the participants. Most of the participants were male (66.2%), and their ages ranged from 34 to 94 years (68.5 ± 12.5). Regarding the health history information, most participants had a family history of CHD (58.9%) and experienced ST elevation, acute myocardial infarction (STEMI) or non-STEMI. In addition, 90.9% of the patients needed to undergo some form of surgery (CABG, PCI, Stent) to treat cardiac complications. Furthermore, most participants had a history of diabetes (62.9%), dyslipidemia (82.5%), heart failure (56%) and peripheral arterial disease (24.4%). Also, there was a difference in anxiety levels between the first (99.2%) and second (80.3%) measurements. Lastly, only 2.2% of the participants called the nurse consultant between the first and second measurement.

Table 2 presents the behavioral and physiological changes pre- and post-intervention. There were significant changes in psychological variables (anxiety) and behavioral variables (BMI, weight, smoking, alcohol, physical activity, family engagement and adherence to the care plan).

Table 3 illustrates the changes pre–post-intervention concerning laboratory results (tests). There were statistically significant changes in reductions in urea, blood sugar, uric acid, total cholesterol, HDL, LDL, triglycerides, AST and ALT.

Table 4 shows the effectiveness of psychological and behavioral variables on adherence to care plans using binary outcomes (adherence vs. non-adherence). A binomial distribution with a logic link was specified for the binary outcome. Specifically, our analyses showed that higher anxiety levels were significantly associated with lower odds of adherence (coefficient = −0.10, SE = 0.02, *p* < 0.001). Participants with a diagnosis of dyslipidemia presented with significantly increased adherence to a care plan (beta 0.82, *p* = 0.02) compared to those with no dyslipidemia, whereas participants with a family history of CHD (beta −0.71, *p* = 0.04) presented with significantly decreased adherence to a care plan. No other significant associations were observed.

Table 5 shows the differences between B-blockers and pulse rates at the first and second measurements.

## 4. Discussion

To our knowledge, this is the first study to evaluate a structured nurse-led post-CHD support program and the role of specialist nurses in the context of secondary health prevention in Greece. Our data analysis revealed that participants with higher levels of anxiety showed significantly lower adherence to a care plan, including those with a family history of CHD. Although total cholesterol, obesity, smoking and adherence to a care plan significantly differed between the first and the second measurements (in a single correlation), these variables were not significant predictors in the evaluation of our nurse-led post-CHD support program. Along the same lines, physical activity, alcohol consumption and family engagement were also not significant predictors affecting adherence to care plans, even after adjusting for age, sex and a history of CHD.

The main finding of the present study was that higher anxiety levels were associated with lower adherence to a care plan, suggesting that participants with lower anxiety show greater adherence to care plans. Thus, anxiety during post-CHD rehabilitation may significantly affect adherence to care plans. Most likely, anxiety is common in patients with coronary heart disease and may significantly influence cardiac health [35]. Along the same lines, a study conducted in China showed similar results as regards anxiety, reporting that counseling by a clinician qualified in psychological therapies and counseling significantly reduces anxiety symptoms [36]. Several potential mechanisms might help to explain the adverse association between anxiety and CHD. Anxiety has been associated with decreased heart rate variability [37] and risk of ventricular arrhythmias [38]. In addition, there is a paucity of research regarding this issue, since most studies have revealed the association between anxiety and adherence, with most focusing on the entire medication regimen in patients with cardiovascular diseases [39].

We also found that participants with dyslipidemia showed greater adherence to a care plan compared to those with no dyslipidemia. This may be explained by the impact of our structured nurse-led supportive program on CHD patients. For instance, our participants may have benefited from a healthy diet. Indeed, in this study, dietary factors such as urea, blood sugar, uric acid, total cholesterol, HDL, LDL, triglycerides, AST and ALT were significantly lower at the second measurement. In agreement with our findings, similar results indicate that there is an inverse correlation between HDL-C and CHD [40], that urea is a predictor of CHD outcomes [41] and that high serum AST and ALT are biochemical markers that can be used to predict the severity of CHD and are also independent risk factors of CHD [40]. Findings from other studies have also revealed that there is a direct and strong link between mortality and blood glucose in patients in the chronic phase of any form of cardiovascular disease [42], that elevated serum uric acid levels are associated with cardiovascular diseases and that adults with elevated TG levels have higher CHD risk [43]. Indeed, poor adherence to healthcare recommendations, medications and treatments is directly related to poorer outcomes, lower quality of life and higher healthcare costs [44].

Another important finding was the differences between the two measurements of behavioral factors (BMI, weight, smoking, alcohol, physical activity, family engagement and adherence to the care plan). A study in Korea confirmed that the major aspects of cardiac rehabilitation for patients with CHD are drug administration, diet, exercise, smoking cessation and stress management, for which the existing health behaviors of patients in their everyday lives need to change [45]. Therefore, practicing health behaviors is a major factor in preventing recurrence after percutaneous coronary interventions for secondary prevention [46].

Finally, this study also demonstrated statistically significant differences between B-blockers and pulse rates. B-blockers should be used as a first-line therapy for symptom relief in patients with stable angina in CHD to relieve ischemia and angina primarily because of negative inotropic and chronotropic action [47]. Many clinical trials have been conducted on beta-blockers, which have been shown to prolong life in patients with cardiovascular disease [48]. Although beta-blockers are a broad class of medications that are used for various clinical benefits, there is a high risk for adverse effects. Therefore, nurses will generally be the first healthcare professionals to take note of any unwanted effects, such as a change in vital signs [49]. When a patient is admitted to an inpatient ward, monitoring clinical effects and potential adverse effects is an interprofessional task. This is crucial because excessively high serum levels can have serious or even fatal consequences [50].

It is true that nursing has a purely humanitarian side, but it also deals with patients as separate human entities [51]. In the present research, a specific nurse-led program was constructed. It was the first attempt at such a study in Greece. The results showed that nurses provide a significant contribution regarding the behavioral and psychological aspects of CHD.

### Future Implications

This study addresses an important gap in the effectiveness of nurse-led post-CHD programs by providing new insights into the important role of nurses in secondary prevention. In single-group studies, to ensure that interventions are well organized and effective, emphasis needs to be placed on adherence to care plans. Nurses can play an important role in uncovering reasons for nonadherence and working with patients to meet goals that are important and relevant to them [52]. Three key factors are important to ensure adherence to a nursing care plan: first, clear and frequent communication among the care team members [53]. This includes sharing relevant information, feedback and updates on the patient or client’s condition, progress and preferences. The second factor is educating and empowering the patient or client by providing them with accurate, relevant and understandable information about their condition; the third factor is monitoring and evaluating the implementation of the program and outcomes by collecting and analyzing data on the patient or client’s condition and progress. Additionally, the shortage of nursing staff and the job dissatisfaction among Greek nurses, who hope to leave their current jobs, make this situation more pressing. Under these circumstances, it is challenging for nurses to be able to turn this crisis into an opportunity for the mutual benefit of nurses, patients and the healthcare system. The government needs to reform and establish advanced roles for nurses, such as by providing a “CHD specialty”, to provide nurses with an appropriate level of autonomy and authority.

## 5. Limitations

Although the present study has offered important findings, it has a few limitations. In this prospective follow-up study, the main limitation was the absence of a control group; thus, this approach did not allow us to draw inferences about the causal relationships between and among the variables. Indeed, we recognized the use of a control group, such as in a Randomized Control Trial (RCT), as the most relevant methodological approach; however, participants enrolled in RCTs may or may not adequately represent the full population which this study was designed to represent [21]. In addition, RCTs evaluate the effects of treatments at population levels and does not explain why outcomes were more effective with those interventions [54]. Another potential limitation could be that the sample collection was performed by a single hospital and therefore generalizations cannot be made. However, we performed repeated measures to eliminate potential collection bias. It would be preferable if the sample had been collected from more centers in the country. Another limitation could also be the reliability of data, as some of the data were based on participants’ self-reports, which may increase the degree of bias and lead to unsafe conclusions. Nevertheless, most of the variables involved laboratory tests. Moreover, an element that could increase the credibility of the conclusions of our research even more would be the possibility of carrying out more than two separate meetings with each patient.

## 6. Conclusions

Our findings indicate that the nurse-led post-CHD support program was found to be partially feasible and effective in our single-group study, emphasizing the need for effective training and retention strategies to enhance the specialization of nurses providing post-CHD support. They also revealed that participants adhering to nurse-tailored care plans presented lower anxiety, suggesting that the active participation of the specialized nurse in secondary prevention is crucially meaningful and can be decisive in helping patients with CHD. The studied impact seems positive, and emphasis should be placed on providing nurses with advanced training and the role of communicating with patients and their relatives. It is obvious, therefore, that preventive strategies targeting well-designed approaches (structured visit notes, interventions, etc.) and post-CHD care management by a trained and qualified nurse collaborating with CHD clinicians with a special focus on secondary prevention are crucially important.

## Figures and Tables

**Table 1:** Demographic and health history characteristics of the study group (*n* = 275).

Demographic Characteristics	*Min*	*Max*	*M (SD)*
Age	34	94	68.5 ± (12.5)
	**Group**	***Frequency***	***Percentage%***
Sex	Male	182	66.2
	Female	93	33.8
**Health History Characteristics**	**Group**	***Frequency***	***Percentage%***
Family History of CHD	No	113	41.1
	Yes	162	58.9
Coronary Artery Disease	Stable Angina	67	24.4
	Unstable Angina	45	16.4
	AΜΙ STEMI	91	33.1
	AΜΙ NSTEMI	72	26.2
Intervention (CABG, PCI, STENT)	No	25	9.1
	Yes	250	90.9
Diabetes	No	173	62.9
	Yes	102	37.1
Dyslipidemia	No	48	17.5
	Yes	227	82.5
	**Group**	***Frequency***	***Percentage%***
Atrial Fibrillation	No	210	76.4
	Yes	65	23.6
Heart Failure	No	154	56.0
	Yes	121	44.0
Peripheral Arterial Disease	No	208	75.6
	Yes	67	24.4
Anxiety First Measurement	No	2	0.7
	Yes	273	99.2
Anxiety Second Measurement	No	54	19.6
	Yes	221	80.3
Call the Nurse Consultant between First and Second Measurement	No	269	97.8
	Yes	6	2.2

Abbreviations: AΜΙ STEMI: acute myocardial infarction ST-segment elevation myocardial infarction; AΜΙ NSTEMI: acute myocardial infarction non-ST-segment elevation myocardial infarction; CABG: coronary artery bypass graft; PCI: percutaneous coronary intervention; M: mean value; SD: standard deviation.

**Table 2:** Behavioral and physiological changes pre- and post-intervention (n = 275).

		*First Measurements*		*Second Measurements*			
***Psychological*** ***Measurements***		***N***	***M (SD)***	***N***	***M (SD)***	***%*** ***Mean Difference***	***p***
Anxiety	Within normal range	2	42.50 (1.50)	54	41.98 (1.80)	−1.22%	0.03 *
	Minimal to moderate	112	54.27 (4.07)	164	49.63 (3.56)	−8.55%	
	Moderate to severe	161	64.11 (3.40)	48	67.60 (4.22)	+5.44%	
	Severe	-	-	9	76.89 (2.71)	-	
*Behavioral* *Measurements*		***N***	***M (SD)***	***N***	***M (SD)***	***% Mean Difference***	***p***
BMI	Normal	53	22.75 (1.70)	79	23.01 (1.44)	+1.14%	0.00 **
	Overweight	128	27.52 (1.31)	131	26.90 (1.23)	−2.25%	
	Obesity	94	33.69 (3.12)	65	32.79 (2.47)	−2.67%	
Weight	Kilos	275	86.17 (17.85)	275	81.63 (15.71)	−5.27%	0.00 **
		***N***	***%***	***N***	***%***	***% Difference***	***p***
Smoking	Never	68	24.73	68	24.73	0	0.00 **
	Former	91	33.09	145	52.72	+19.63%	
	Current	116	42.18	62	22.55	−19.63%	
Alcohol consumption	No	181	65.80	197	71.63	+5.83%	0.00 **
	Yes	94	34.20	78	28.37	−5.83%	
Physical activity	No	216	78.55	211	76.73	−1.82%	0.19
	Yes	59	21.45	64	23.27	+1.82%	
Family engagement	No	131	47.64	96	34.90	−12.74%	0.00 **
	Yes	144	52.36	179	65.10	+12.74%	
Adherence to care plan	No	58	21.10	30	10.90	−10.20%	0.00 **
	Yes	217	78.90	245	89.10	+10.20%	

Abbreviations: BMI: Body Mass Index; M: mean value; SD: Standard Deviation; N: number of cases; Note: ** Significance at level *p* < 0.01; * Significance at level *p* < 0.05.

**Table 3:** Measurements and changes in variables pre- and post-intervention (n = 275).

	*First Measurement*		*Second Measurement*			
*Dietary Factors*	*M (SD)*	*Min–Max*	*M (SD)*	*Min–Max*	*Paired Mean Difference (SD)*	*p*
Urea	44.83 (24.74)	4–267	40.9 (19.44)	11–213	3.93 (12.28)	0.000 **
Serum Glucose	115.25 (39.64)	63–371	102.9 (25.84)	43–269	12.36 (28.71)	0.000 **
Uric Acid	5.43 (2.8)	2.3 -12	4.88 (1.11)	2.1–9.3	0.54 (2.55)	0.000 **
Total Cholesterol	163.27 (48.44)	62–320	134.44 (40.99)	71–287	28.83 (38.89)	0.000 **
HDL	49.62 (27.41)	20–107	55.29 (12.97)	21–87	−5.67 (26.48)	0.000 **
LDL	102.92 (37.51)	40–273	90.16 (30.68)	39–291	12.76 (25.17)	0.000 **
Triglycerides	124.01 (63.66)	45–492	106.77 (43.62)	41–367	17.24 (42.75)	0.000 **
AST	32.19 (32.51)	6–164	23.61 (12.36)	7–93	8.58 (29.96)	0.000 **
ALT	29.29 (15.98)	2–96	24.27 (16.02)	7–140	5.02 (17.81)	0.000 **

Abbreviations: HDL: high-density lipoprotein; LDL: low-density lipoprotein; AST: aspartate transaminase; ALT: alanine transaminase; M: mean value; SD: Standard Deviation; Note: ** Significance at level *p* < 0.01.

**Table 4:** Effect of psychological and behavioral interventions on adherence to care plan (n = 275).

	*β*	*SE*	Wald	*p*
**Intercept (*α*)**	10.88	2.00	29.58	0.00 **
Anxiety	−0.10	0.02	28.01	0.00 **
BMI	−0.09	0.06	2.46	0.12
Weight	−0.01	0.02	0.32	0.57
Smoking	0.08	0.34	0.06	0.69
Physical activity	0.73	0.42	3.03	0.10
Alcohol consumption	0.09	0.34	0.06	0.80
Family engagement	−0.10	0.29	0.09	0.76
Adjustment variables				
Sex (male vs. female)	−0.42	0.42	0.99	0.32
Age (years)	−0.01	0.01	0.05	0.83
Dyslipidemia (yes vs. no)	0.94	0.38	6.22	0.01 **
Family history of CHD (yes vs. no)	−0.72	0.35	4.29	0.04 *

Abbreviations: SE: standard error; β: beta; BMI: Body Mass Index; GEE: Generalized Estimating Equation. Notes: Methods: Repeated Measures analysis, logistic regression via generalized estimating equations (GEEs); adherence to plan care set as outcome variable; Anxiety, BMI, Weight, Smoking, Alcohol consumption, Family engagement as predictor variables; sex, age, dyslipidemia and Family History of CHD as adjustment variables. ** Significance at level *p* < 0.01; * Significance at level *p* < 0.05.

**Table 5:** Independent samples *t*-test comparison of pulse rate measurements between patients receiving/not receiving B-blockers.

	*M (SD)*	*M (SD)*	*t (275)*	*p*
***Pulse Rate × B-Blockers***	*B-Blockers {YES}*	*B-Blockers {NO}*		
	*5.86 (7.50)*	*4.04 (6.50)*	*−2.12*	0.03 *
***Pulse Rate × Cardioselective B-Blockers***	*Cardioselective {YES}*	*Cardioselective {NO}*		
	*6.34 (7.99)*	*4.18 (6.36)*	*−2.47*	0.01 **
***Pulse Rate × Cardioselective B-Blockers***	*Non* *Cardioselective*	*Non Cardioselective*		
	*{YES}*	*{NO}*		
	*4.61 (5.96)*	*5.09 (7.29)*	*0.39*	0.69 **

** Significance at level *p* < 0.01; * Significance at level *p* < 0.05.

## Data Availability

The data presented in this study are available on request from the corresponding author(s). The data are not publicly available due to privacy restrictions.
